# Leukoaraiosis Mediates the Association of Total White Blood Cell Count With Post-Stroke Cognitive Impairment

**DOI:** 10.3389/fneur.2021.793435

**Published:** 2022-02-03

**Authors:** Wanying Shan, Liang Xu, Yuan Xu, Zhuoyin Qiu, Jie Feng, Jie Zhao, Jingwen Wang

**Affiliations:** ^1^Department of Neurology, Suzhou Ninth People's Hospital, Soochow University, Suzhou, China; ^2^Department of Anesthesiology, The First Affiliated Hospital of Soochow University, Suzhou, China; ^3^Department of Gerontology, Suzhou Ninth People's Hospital, Soochow University, Suzhou, China

**Keywords:** leukoaraiosis, white blood cell, PSCI, mediation analysis, stroke

## Abstract

**Background and Purpose:**

The inflammatory response could play a key role in cognitive impairment. However, there has been limited research into the association between total white blood cell (WBC) count and post-stroke cognitive impairment (PSCI), and the significance of leukoaraiosis (LA) in this relationship is unknown. We aimed to examine the total WBC count in relation to PSCI and whether this association was mediated by LA.

**Methods:**

Consecutive patients with first-ever ischemic stroke were prospectively enrolled from October 2020 to June 2021. The total WBC count was measured after admission. Cognitive function evaluations were performed at the 3-month follow-up using Mini-mental State Examination (MMSE). We defined the PSCI as an MMSE score <27.

**Results:**

A total of 276 patients (mean age, 66.5 years; 54.7% male) were included in this analysis. Among them, 137 (49.6%) patients experienced PSCI. After adjustment for potential confounders, higher total WBC count was significantly correlated with an increased risk of LA [per 1-SD increase, odds ratio (*OR*), 1.39; 95% *CI* 1.06–1.82; *p* = 0.017] and PSCI (per 1-SD increase, *OR*, 1.51; 95% *CI* 1.12–2.04; *p* = 0.006). Furthermore, mediation analysis demonstrated that the association between total WBC count and PSCI was partly mediated by LA (the regression coefficient was changed by 9.7% for PSCI, and 12.4% for PSCI severity, respectively).

**Conclusion:**

Increased total WBC count is a risk factor for PSCI. The presence of LA was partially responsible for the PSCI in patients who had a higher total WBC count.

## Introduction

In China, ischemic stroke has been ranked as the first leading cause of major disability and death (1). Post-stroke cognitive impairment (PSCI) is the most common complication with a prevalence ranging from 20 to 80% (2). It involves deficits in the ability to think and remember, language, and attention that are significant enough to greatly impact daily life (3). PSCI is reported to influence the performance in daily life and at work (4) and increase the risk of long-term mortality (5). Many clinical characteristics have been linked to the advancement of cognitive impairment following a stroke, such as advanced age, female gender, education, and vascular comorbidities (2). However, the underlying mechanisms of PSCI are still unclear. Determining the exact biomarkers and potential mechanisms for PSCI is of vital importance for continuously improving the prognosis of patients with ischemic stroke.

The inflammatory response has been linked to cognitive impairment after ischemic stroke (6–9). Increased total WBC count is associated with adverse clinical outcomes in patients with acute ischemic stroke (10). In addition, a higher WBC count, even within the normal range, was found to be related to the worse psychomotor cognitive performance in the elderly (11). However, the influence of total WBC count on PSCI has not yet been clarified. Leukoaraiosis (LA) is one of the leading risk factors for vascular cognitive impairment (12), and multiple lines of evidence from clinical trials and animal studies indicated that inflammatory response may contribute to the presence and development of LA (13–17). Therefore, LA may be a mediator in the pathway between inflammation and the presence of PSCI.

We, therefore, performed this prospective study to examine: (1) the cross-sectional relationship between increased total WBC count and risk of 90-day PSCI among Chinese patients with ischemic stroke; (2) the mediating effect of LA on this potential relationship.

## Methods

### Study Patients

This prospective study enrolled first-ever acute ischemic stroke from October 2020 to June 2021 in Suzhou Ninth People's Hospital. The inclusion criteria were as follows: (1) age >18 years; (2) the diagnosis of ischemic stroke was confirmed by MRI or CT; and (3) time from stroke onset to hospitalization <7 days. The inclusion criteria were as follows: (1) pre-existed cognitive dysfunction, such as Alzheimer's disease, Parkinson's disease, and any psychiatric illness; (2) had an infectious disease within 1 month before stroke onset; (3) unable to perform the cognitive function assessment due to severe neurological deficit; (4) life expectancy <3 months; and (5) had a history of central nervous system disease, renal failure, hepatic failure, autoimmune disease, and thyroid hormone disorders. The Suzhou Ninth People's Hospital's Ethical Committee approved the study protocol. The patients signed a written informed consent before entering the study.

### Data Collection

Data on demographics and clinical factors were recorded after admission. Basic information included age, gender, and education year. Traditional vascular risk factors that were recorded included hypertension (defined as presenting with a history of hypertension or being treated with antihypertensive medication), diabetes mellitus (defined as fasting plasma glucose ≥7.0 mmol/L, or 2-h postprandial glucose ≥11.1 mmol/L, or the use of insulin/oral hypoglycemic medication), hyperlipidemia (defined as total cholesterol ≥5.2 mmol/L, or low-density lipoprotein cholesterol ≥2.6 mmol/L, or triglyceride ≥1.70 mmol/L, or being treated with lipid-lowering medicines), ischemic heart disease (defined as a history of myocardial infarction or angina pectoris), and current smoker (18). The severity of ischemic stroke was evaluated by a trained neurologist using the National Institutes of Health Stroke Scale (NIHSS) (19). The stroke etiology was determined using the Trial of Org 10172 in Acute Stroke Treatment criteria (20). Laboratory data, such as total WBC count, platelet count, lipid profile, fasting blood-glucose, homocysteine, and high-sensitivity C-reactive protein (Hs-CRP) levels were all measured within 24 h after admission.

### Imaging Review and Analysis

All patients were performed CT or MRI within the 7 days after admission. Neuroimaging was reviewed independently by two readers who were blinded to clinical data. The infarction volume was assessed by the DWI-based Alberta Stroke Program Early CT Score (DWI-ASPECTS). LA was defined as patchy or diffuse areas of hypodensity on CT or hyperintensity on T2-weighted MRI in periventricular or subcortical regions, or in the pons (21). In case of disagreement, lesions were determined by consensus. For the diagnosis of LA, limited intra-rater reliability testing revealed good reliability with a kappa value of 0.86.

### Cognitive Function Assessment

All participants were followed up at 3 months by trained neurologists. The Mini-mental State Examination (MMSE) was performed to evaluate the cognitive function in this study. According to previous literature, the PSCI was defined as an MMSE score of <27 (22–24). Moreover, the PSCI severity was categorized as follows: 0–22 (severe PSCI), 23–26 (mild PSCI), and 27–30 (no PSCI).

### Statistical Analysis

Summaries of categorical variables are reported as proportions, and continuous variables as means ± SD or medians (interquartile ranges [IQR]). We analyzed the differences between the groups using Fisher's exact test or the χ^2^ test for categorical variables, and one-way ANOVA or Kruskal–Wallis test for continuous variables. Associations of WBC count with LA and PSCI were evaluated using binary logistic regression. We estimated adjusted odds ratios (*OR*s) and their 95% *CI*. All multivariable analyses were first adjusted for age, sex, and education years (Model 1) and additionally adjusted for all variables with a *p* < 0.1 in the univariate analysis (such as, age, sex, education years, diabetes mellitus, baseline NIHSS score, DWI-ASPECTS 0–7, uric acid, and Hs-CRP level; Model 2). The effect of total WBC count on PSCI severity was analyzed using ordinal logistic regression models. In addition, to determine whether LA could mediate the effect of total WBC count on PSCI, the mediation analysis was performed to calculate the proportion mediated and evaluate its significance by the Sobel test.

Statistical analysis was performed using the SPSS version 24.0 software package (SPSS Inc, Chicago, IL, USA), and 2-tailed values of *p* < 0.05 were considered statistically significant.

## Result

A total of 276 patients (mean age, 66.5 years; 54.7% male) were recruited for the analysis. Among these patients, 66.3% had hypertension, 27.9% had diabetes, and 14.9% had hyperlipidemia. The median total WBC count was 6.4 × 10^9^/L. General and clinical data stratified by subjects with and without PSCI are presented in (Table 1). As compared with patients without PSCI, those with it were older, had a greater prevalence of diabetes mellitus, education years <12, and LA, and had higher baseline NIHSS score, WBC count, uric acid, and Hs-CRP level.

**Table 1:** Baseline data stratified by patients with and without PSCI.

**Variables**	**All patients, *n* = 276**	**With PSCI, *n* = 137**	**Without PSCI, *n* = 139**	***P*-value**
Age, years	66.5 ± 9.1	68.6 ± 8.3	64.4 ± 9.4	0.001
Male, *n* (%)	151 (54.7)	70 (51.1)	81 (58.3)	0.231
Education years <12, *n* (%)	174 (63.0)	99 (72.3)	75 (54.0)	0.002
Risk factors, *n* (%)				
Hypertension	183 (66.3)	96 (70.1)	52 (62.6)	0.189
Diabetes mellitus	77 (27.9)	52 (38.0)	25 (18.0)	0.001
Hyperlipidemia	41 (14.9)	21 (15.3)	20 (14.4)	0.826
Coronary heart disease	34 (12.3)	17 (12.4)	17 (12.2)	0.964
Current smoker	102 (37.0)	50 (36.5)	52 (37.4)	0.875
Clinical data				
Time from onset to hospitalization, days	3.0 (1.0, 4.0)	3.0 (2.0, 4.0)	3.0 (1.0, 4.0)	0.102
Systolic blood pressure, mmHg	138.3 ± 16.8	138.1 ± 15.9	138.5 ± 17.6	0.840
Diastolic blood pressure, mmHg	80.8 ± 10.3	80.8 ± 9.5	80.7 ± 10.9	0.891
Baseline NIHSS, score	5.0 (3.0, 8.0)	5.0 (3.0, 8.0)	5.0 (2.0, 7.0)	0.008
DWI-ASPECTS 0–7, *n* (%)	122 (46.9)	68 (53.2)	54 (41.5)	0.082
Leukoaraiosis, *n* (%)	115 (41.7)	68 (49.6)	47 (33.8)	0.008
Stroke etiology, *n* (%)				0.561
Large-artery disease	120 (43.5)	55 (40.1)	65 (46.8)	
Small-artery disease	77 (27.9)	31 (22.6)	23 (16.5)	
Cardioembolic stroke	54 (19.6)	38 (27.7)	39 (28.1)	
Others and undetermined	25 (9.1)	13 (9.5)	12 (8.6)	
Laboratory data				
Platelet count, 10^9^/L	206.7 ± 74.5	205.0 ± 64.5	208.3 ± 83.4	0.717
Homocysteine, mmol/L	14.2 ± 6.1	14.1 ± 6.6	14.4 ± 5.1	0.690
Hs-CRP, mg/L	5.8 (2.8, 10.0)	7.6 (3.3, 10.5)	5.2 (2.4, 9.7)	0.001
Fasting blood-glucose, mmol/L	5.9 ± 2.5	6.1 ± 2.6	5.8 ± 2.5	0.366
Uric acid, umol/l	309.6 ± 103.6	323.3 ± 113.2	296.2 ± 91.7	0.031
Total cholesterol, mmol/L	4.2 ± 1.1	4.3 ± 1.2	4.2 ± 1.1	0.438
Triglyceride, mmol/L	1.4 (1.0, 1.8)	1.4 (1.0, 1.8)	1.3 (1.3, 1.8)	0.738
Low density lipoprotein, mmol/L	2.4 (2.0, 2.9)	2.4 (1.9, 2.9)	2.3 (2.0, 3.1)	0.515
High density lipoprotein, mmol/L	1.0 (0.9, 1.1)	1.1 (0.9, 1.2)	1.0 (0.8, 1.2)	0.469
Total WBC count, 10^9^/L	6.6 ± 2.0	7.1 ± 2.2	6.1 ± 1.6	0.001
Total WBC count quartile				0.003
First quartile	69 (25.0)	24 (17.5)	45 (32.4)	
Second quartile	71 (25.7)	33 (24.1)	38 (27.3)	
Third quartile	68 (24.6)	35 (25.5)	33 (23.7)	
Fourth quartile	68 (24.6)	45 (32.8)	23 (16.5)	

*DWI-ASPECTS, DWI based alberta stroke program early CT score; Hs-CRP, high-sensitivity C-reactive protein; LA, leukoaraiosis; NIHSS, national institutes of health stroke scale; PSCI, post-stroke cognitive impairment; WBC, white blood cell*.

Table 2 summarizes the results of the binary logistic regression of the LA. Participants in the fourth quartile of total WBC count had a substantially higher risk of LA (*OR*, 2.67; 95% *CI* 1.34–5.33; *p* = 0.005) than those in the first quartile, according to a univariate logistic regression analysis. The risk was slightly attenuated but persisted after adjustments for age, sex, education years, diabetes mellitus, baseline NIHSS score, DWI-ASPECTS 0–7, uric acid, and Hs-CRP level (*OR*, 2.00; 95% *CI* 1.03–4.20; *p* = 0.046). When the total WBC count was included as a continuous variable, the results were similar (per 1-SD increase, *OR*, 1.39; 95% *CI* 1.06–1.82; *p* = 0.017).

**Table 2:** Multivariate logistic regression analysis for the association between total WBC count and leukoaraiosis.

**Variables**	**Crude model**		**Model 1**		**Model 2**	
	**OR (95% CI)**	***P*-value**	**OR (95% CI)**	***P*-value**	**OR (95% CI)**	***P*-value**
Total WBC count, per SD increase	1.55 (1.21–1.99)	0.001	1.51 (1.18–1.94)	0.001	1.39 (1.06–1.82)	0.017
Total WBC count						
First quartile	Reference		Reference		Reference	
Second quartile	0.95 (0.48–1.91)	0.900	0.94 (0.47–1.89)	0.871	0.81 (0.38–1.69)	0.570
Third quartile	0.96 (0.48–1.93)	0.909	0.93 (0.46–1.90)	0.851	0.75 (0.34–1.62)	0.464
Fourth quartile	2.67 (1.34–5.33)	0.005	2.41 (1.20–4.87)	0.014	2.00 (1.03–4.20)	0.046

*CI, confidence interval; OR, odds ratio; WBC, white blood cell*.

*Model 1: adjusted for age and sex*.

*Model 2: adjusted for age, sex, education years, diabetes mellitus, baseline NIHSS score, DWI-ASPECTS 0–7, uric acid and Hs- CRP level*.

**Figure 1:**
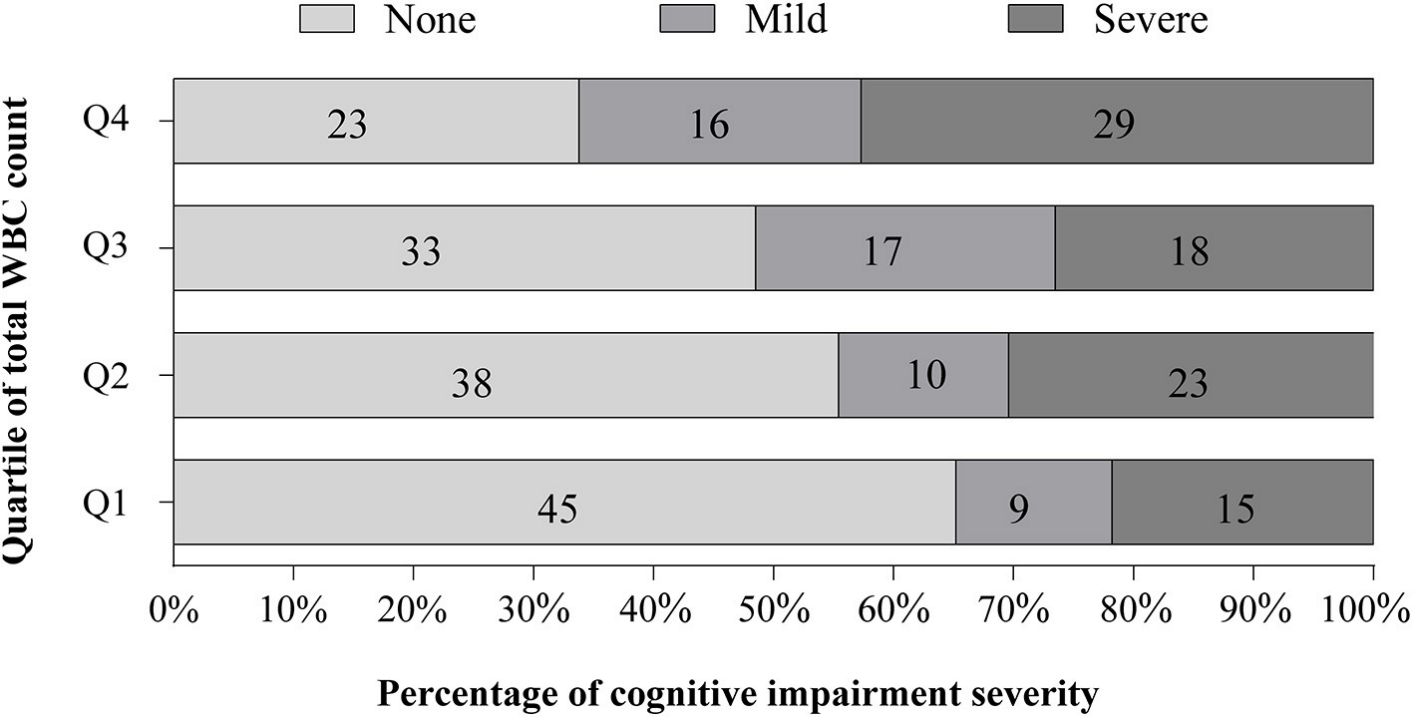
Distribution of cognitive impairment severity at 90 days stratified by the quartile of total white blood cell (WBC) count. There was a significant difference between total WBC count in the overall distribution of severity of post-stroke cognitive impairment (PSCI) (*p* = 0.012). After adjustment for the same variable in model 2, the ordinal regression analyses showed a significant association between total WBC count and PSCI severity [fourth quartile vs. first quartile, odd ratio (*OR*), 3.25; 95% *CI* 1.68–6.27, *p* < 0.001].

At the 3 months follow-up, 137 (49.6%) patients had developed PSCI. As shown in (Table 3), we found that total WBC count was associated with an increased risk of PSCI after adjustment for potential confounders. Compared with subjects in the first quartile of total WBC counts, those in the fourth quartile had a 149% increased risk of PSCI (*OR*, 2.49; 95% *CI* 1.11–5.56; *p* = 0.026). According to the MMSE score categories, 52 (18.8%) participants had mild PSCI and 85 (30.8%) had severe PSCI. Ordinal regression analyses showed a significant relationship between the total WBC count and PSCI severity (highest quartile vs. lowest quartile, *OR* 3.25; 95% *CI* 1.68–6.27, *p* < 0.001; Figure 1).

**Table 3:** Multivariate logistic regression analysis for the association between total WBC count and PSCI.

**Variables**	**Crude model**		**Model 1**		**Model 2**	
	**OR (95% CI)**	***P*-value**	**OR (95% CI)**	***P*-value**	**OR (95% CI)**	***P*-value**
Total WBC count, per SD increase	1.62 (1.25–2.10)	<0.001	1.57 (1.20–2.05)	0.001	1.51 (1.12–2.04)	0.006
Total WBC count						
First quartile	Reference		Reference		Reference	
Second quartile	1.63 (0.83–3.22)	0.160	1.71 (0.85–3.49)	0.135	1.60 (0.75–3.43)	0.226
Third quartile	1.99 (1.00–3.95)	0.050	2.25 (1.09–4.63)	0.029	2.16 (0.93–4.78)	0.059
Fourth quartile	3.67 (1.81–7.43)	<0.001	3.29 (1.57–6.92)	0.002	2.49 (1.11–5.56)	0.026

*CI, confidence interval; OR, odds ratio; PSCI, post-stroke cognitive impairment; WBC, white blood cell*.

*Model 1: adjusted for age and sex*.

*Model 2: adjusted for age, sex, education years, diabetes mellitus, baseline NIHSS score, DWI-ASPECTS 0–7, uric acid and Hs- CRP level*.

**Figure 2:**
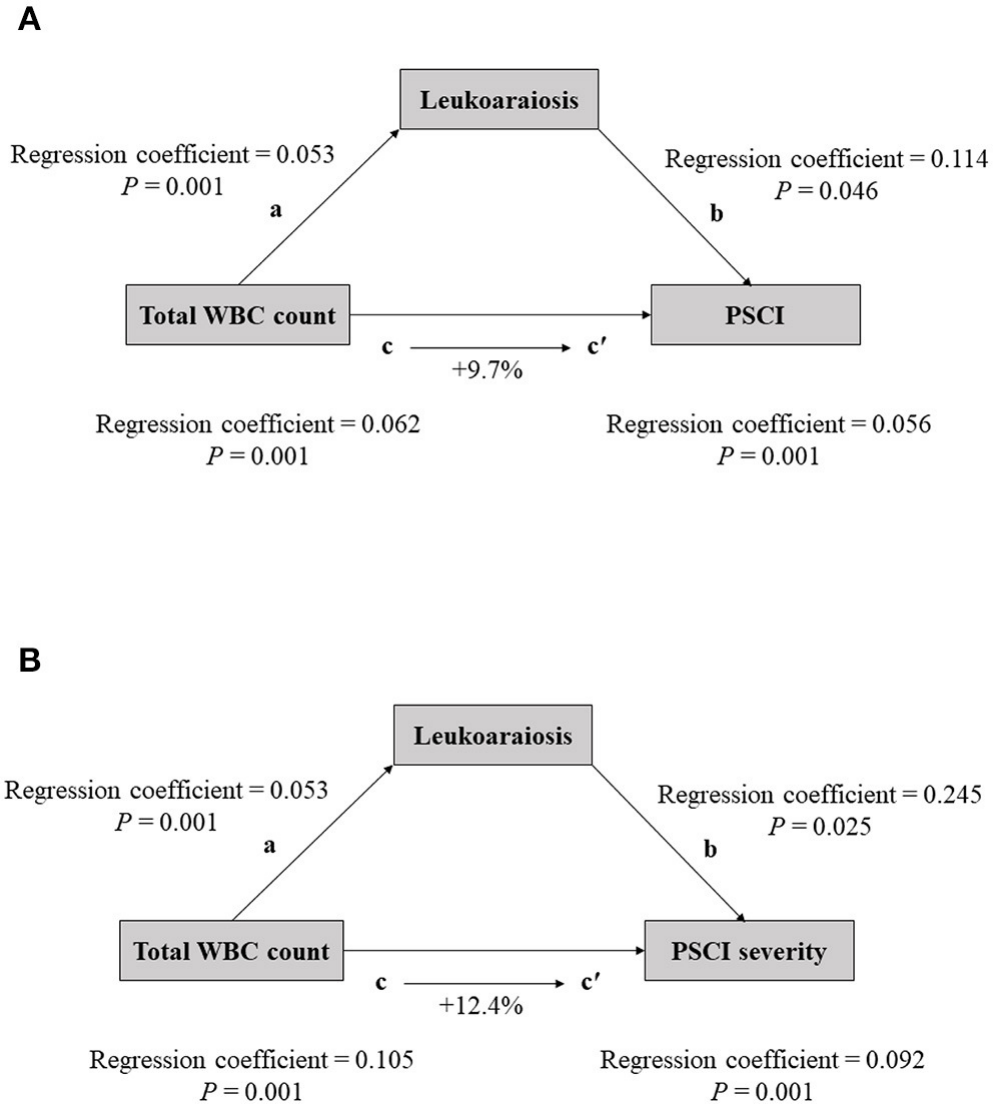
The mediation analysis by leukoaraiosis (LA) of the association between the total WBC count and cognitive impairment [**(A)** for PSCI; **(B)** for PSCI severity]. (a) Regression coefficient of the association between total WBC count and cognitive impairment; (b) the regression coefficient of the association between LA and cognitive impairment, using total WBC count and LA as independent variables; (c) regression coefficient of the association between total WBC count and cognitive impairment; (c′) regression coefficient of the association between total WBC count and cognitive impairment, using total WBC count and leukoaraiosis as independent variables. The percentage difference of the coefficients (1–c/c′) is shown.

We next evaluated the effect of LA on the association between total WBC count and PSCI. Mediation analysis showed that LA was a mediator of the effect of total WBC count on 3-month PSCI (9.7% mediation for PSCI, and 12.4% mediation for PSCI severity, Figure 2).

## Discussion

Our prospective investigation discovered a link between the total WBC count and the presence and severity of PSCI. We performed mediation studies, which revealed that LA was a major mediator of the relationship between total WBC count and PSCI.

The total WBC count, a typical indicator of systemic inflammation, is known to be increased in ischemic stroke and to play a role in the severity of the ischemic damage (10). Increase neutrophil count is found to be related to the infarct size and functional outcome in large hemispheric infarction patients (25). However, we did not yield a similar result with prior studies. We found that the link between the total WBC count and infarction volume was not significantly different (*p* = 0.399). This discrepancy might be partly due to the difference in measurement of the infarction volume. The DWI-ASPECT score was employed to estimate the infarct size in this investigation, which is less precise than a fully quantitative method.

Numerous epidemiological studies have indicated that increased total and differential WBC counts, even within the normal range, were significantly correlated with cognitive impairment (11, 26, 27). According to the National Health and Nutrition Examination Survey, a higher total WBC count in the elderly is associated with the lower psychomotor cognitive performance (11). Using a case-control study, An et al. (26) demonstrated that elderly Chinese adults with a neutrophil-lymphocyte ratio higher than the threshold of 2.07 were significantly associated with the risk of mild cognitive impairment. Furthermore, Halazun et al. (27) recruited carotid endarterectomy patients from Columbia University and found that the preoperative neutrophil-lymphocyte ratio >5 is significantly associated with higher odds of cognitive dysfunction. Consistent with prior literature, the current study of 276 patients with acute ischemic stroke showed a negative association between increased total WBC count and PSCI. The detrimental effects of increased total WBC count on cognitive function after ischemic stroke are unclear. One major assumption is that WBC could drive the response of immune system and have a role in boosting MMP-9 levels, which can lead to blood-brain barrier leakage and brain tissue degeneration (28). Moreover, previous studies have suggested that increased numbers and activation of circulating WBC may contribute to organ ischemia by harming the endothelium and increasing oxidative stress (29, 30). Taken together, increased and activated WBC has a role in the pathogenesis of cognitive impairment following ischemic stroke and promotes its progression through multiple mechanisms.

We observed a significant relationship between total WBC count and LA. Several etiologies have been proposed to explain the LA seen in vascular cognitive impairment. Damage to the blood-brain barrier with leakage of inflammatory factors in the white matter has been postulated (16). In addition, WBC count and the presence of LA may be linked by a subclinical inflammation in the vessel wall. Activated WBC might stimulate the cytokines release and the generation of reactive oxygen species (ROS), which may induce further vascular damage (31). The LA is generally observed around blood vessels associated with pathological changes, particularly in the medullary arterioles of the deep white matter (32). Meanwhile, compelling evidence between LA and cognitive impairment in patients with ischemic stroke has been found in previous epidemiological studies (12, 17, 33). LA may induce the loss of microstructural integrity in white matter tracts, and hamper the functional compensation through remote brain areas after stroke (34–36). Therefore, LA, may play a crucial role in the development of PSCI caused by the increased total WBC count. As expected, our study further showed that the indirect effect mediated by LA accounted for 9.7% of the correlation between WBC count and PSCI. Further studies are warranted to elucidate these potential mechanisms.

This study has several limitations that should be acknowledged. First, the study was performed only in one stroke center. Therefore, our findings may not apply to other patients. Second, the total WBC count was only tested during the acute stage of ischemic stroke and, hence, this study yielded no data regarding when and how long WBC count was changed. Third, participants in this study were likely experiencing some form of unidentified systemic inflammation prior to the stroke onset, which could lead to systematic bias. Finally, patients who were unable to complete the cognitive function examinations were excluded from this study, which may have resulted in an underestimation of PCI prevalence. Extrapolating our findings should be done with caution.

In summary, our findings demonstrated that the increased total WBC count is a risk factor for 90-day PSCI in patients with ischemic stroke. The presence of LA was partially responsible for worse cognitive impairment in individuals with a higher total WBC count. To verify these associations and elucidate the exact mechanism of PSCI, further longitudinal investigations are required.

## Data Availability Statement

The raw data supporting the conclusions of this article will be made available by the authors, without undue reservation.

## Ethics Statement

The studies involving human participants were reviewed and approved by Suzhou Ninth People's Hospital. The patients/participants provided their written informed consent to participate in this study.

## Author Contributions

WS, LX, and JW contributed to the conceptualization of the manuscript. WS, LX, YX, and ZQ contributed to data curation. WS, LX, and YX performed the formal analysis. WS, LX, YX, ZQ, and JF contributed to the investigation and methodology. WS, LX, and JZ contributed to the project administration. JZ and JW performed the supervision. LX, YX, and JF are responsible for the validation of manuscript. WS contributed to the writing original draft. YX, JZ, and JW contributed to the writing review and editing of the manuscript. All authors contributed to the article and approved the submitted version.

## Conflict of Interest

The authors declare that the research was conducted in the absence of any commercial or financial relationships that could be construed as a potential conflict of interest.

## Publisher's Note

All claims expressed in this article are solely those of the authors and do not necessarily represent those of their affiliated organizations, or those of the publisher, the editors and the reviewers. Any product that may be evaluated in this article, or claim that may be made by its manufacturer, is not guaranteed or endorsed by the publisher.
